# A Rare Case of Femoral Shaft Fracture Linked to Childbirth

**DOI:** 10.7759/cureus.91954

**Published:** 2025-09-10

**Authors:** Aritra Kapat, Dharmendra Kumar, Nidhi Ranjan, Maheswar Mandi, Koushik Biswas

**Affiliations:** 1 Paediatrics, Ghatal Sub-Divisional Hospital, Ghatal, IND; 2 Biochemistry, All India Institute of Medical Sciences, Raebareli, IND; 3 Paediatrics, Dr. Bidhan Chandra Roy Post Graduate Institute of Paediatric Sciences, Kolkata, IND

**Keywords:** birth injuries, caesarean section, childbirth trauma, femur, femur fractur

## Abstract

Birth injuries occur rarely during Cesarean section thanks to antenatal care, skilled obstetricians, and decreased instrumental maneuvers. Most traumatic fractures during childbirth involve the clavicle and humerus, which are related to malposition and malpresentation. We report a rare case of femoral shaft fracture at childbirth. A 19-year-old primigravida with a normal antenatal history delivered a term newborn with a 2.14 kg birth weight by Cesarean section. A day after birth, the mother noticed a swelling on the baby’s right thigh with mild deviation of the right limb compared to the left one, and excessive crying upon touching the area. Urgent X-ray revealed a closed fracture of the right femur mid-shaft. Conservative management, including splinting and casting, was performed. Follow-up X-ray after three weeks revealed new soft tissue bone formation, and the child was discharged in a hemodynamically stable condition with follow-up advice.

## Introduction

Birth injury is characterized as a structural breakdown of the newborn's body or functional decline caused by mechanical stress during labor, birth, or both. The spectrum of birth injuries includes swelling, bruises, minor cuts and abrasions, compressive nerve injuries, and, in rare cases, fractures. These injuries are often attributed to precipitous labor, malposition, malpresentations, and instrumental deliveries [1]. The most common bone fractures reported at birth involve the clavicle, followed by the humerus. Femoral shaft fracture at birth is very rarely reported in the literature [2]. One study reported the incidence of birth-related femoral shaft fracture to be 0.024 per 1000 live births [3]. Most of the long bone fractures are related to normal vaginal delivery or forceps delivery, with associated risk factors such as macrosomia, multiple pregnancies, extreme prematurity with low birth weight, and existing collagen vascular disease. Whether Cesarean section can lead to a reduction of such birth injuries is a matter of debate [4]. We present the case of a term male baby born to a primigravida mother through emergency Cesarean section who suffered from a fracture of the femoral shaft at birth.

## Case presentation

**Figure 1:**
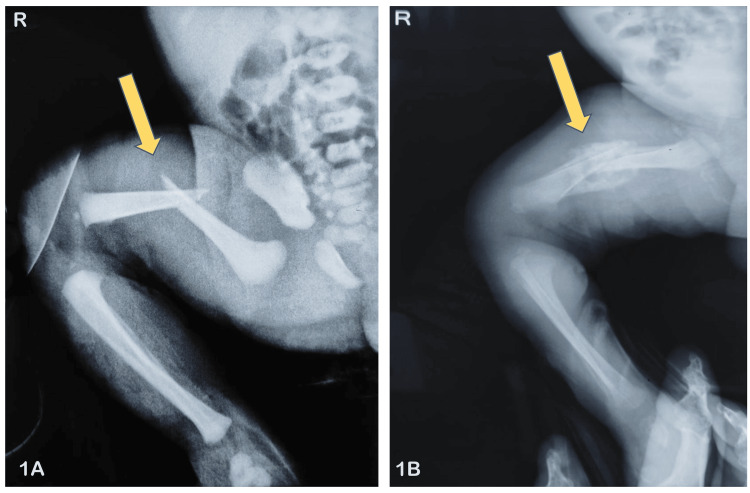
X-ray of the right femur (1A) one day after childbirth and (1B) three weeks after treatment Area of fracture marked with a yellow arrow

Our patient was a male baby born to a 19-year-old primigravida mother via emergency Cesarean section in the Ghatal Sub-Divisional Hospital. The baby was delivered at 37 weeks (as per modified Ballard scoring) with evidence of intrauterine growth retardation, weighing 2.14 kg at birth. The indication for emergency Cesarean section had been oligohydramnios with a decrease in foetal movement. The mother was thin built, primigravida without any history of hypothyroidism, diabetes mellitus, connective tissue disorder, uterine myoma, or evidence of osteoporosis. Her antenatal period had been uneventful without any sign of non-progression to labor. The foetus was in vertex presentation, and the anomaly scan did not reveal any significant congenital anomaly. One day after birth, the mother noticed swelling on the baby’s right thigh with mild deviation of the right limb compared to the left one, and excessive crying on touching the area. An urgent X-ray revealed a closed fracture of the mid-shaft of the femur, type 32-D/5.1 of the right side, as per Arbeitsgemeinschaft für Osteosynthesefragen (AO) Pediatric Comprehensive Classification of Long-bone Fracture (Figure 1A). There was no feature of osteogenesis imperfecta, arthrogryposis, or any other connective tissue disease in the baby. The child's complete blood count and C-reactive protein investigation results done on day three after birth are presented in Table 1.

**Table 1:** Complete blood count and C-reactive protein of the neonate

Parameter		Result	Reference range
Hemoglobin (g/dl)		16.2	14-22
Total leukocyte count (per mm^3^)		16,000	10,000-26,000
Differential count	Neutrophil (%)	48	40-70
	Lymphocyte (%)	42	20-45
	Monocyte (%)	7	1-10
	Eosinophil (%)	3	1-6
	Basophil (%)	0	0-2
Platelet count (per mm^3^)		210,000	150,000 – 450,000
C-reactive protein (mg/dl)		0.42	<0.7

On further investigation, the mother was found to have borderline insufficient 25-hydroxyvitamin D (29 ng/ml) with normal serum calcium (9.2 mg/dl) and serum phosphorus (3.3 mg/dL) levels (Table 2).

**Table 2:** Serum concentration of 25-hydroxyvitamin D, total calcium, and phosphorus in the mother

Parameters	Result	Reference range
25-hydroxyvitamin D (ng/ml)	29.0	30-100
Total calcium (mg/dl)	9.2	8.5-11
Phosphorus (mg/dl)	3.3	2.5-4.5

An orthopedic consultation was sought, and conservative management was advised in the form of strapping the right thigh and leg. While on strapping, the capillary refill time on the right distal toes was thoroughly monitored. A repeat X-ray after three weeks highlighted subperiosteal new bone and callus formation across the fracture site (Figure 1B). The baby was discharged with advice on serial strapping with follow-up X-ray on monthly intervals.

## Discussion

During the series of events of childbirth, any kind of unwanted traction or vigorous manipulation may result in fracture, the clavicle being the most common bone affected. The incidence of birth-related femur shaft fracture is around 0.077 per 1000 live births [4]. The earliest case of a shaft of femur fracture during birth was reported in 1922 following a difficult breech delivery [5]. There is no definitive evidence that Cesarean section reduces the risk of such fractures, and existing literature reports conflicting findings [6,7]. Risk factors for femoral shaft fracture during childbirth include extreme prematurity, low birth weight, malpresentations like breech, shoulder dystocia, macrosomia, abnormal lie, and multiple pregnancy. Hence, the fracture may occur during difficult normal vaginal or forceps delivery.

Other perinatal risk factors associated with this injury include osteogenesis imperfecta, other connective tissue disorders, disuse osteoporosis following prolonged immobilization, congenital bony deformities, and osteopenia of prematurity [5]. In our case, the baby had evidence of mild intrauterine growth retardation and was delivered by emergency Cesarean section. Decreased space for performing obstetric maneuvers, poor uterine relaxation, poor delivery techniques, or a small vertical incision during lower uterine segment Cesarean section may directly or indirectly lead to femoral fractures due to inadvertent sudden torsional traction [8].

No gender predisposition for this condition has been described. In previous sporadic cases and cohorts, a mean duration of 1.5-6.3 days was taken to diagnose the femoral shaft fracture. Paternal neglect, ignorance, or lack of nearby health facilities may lead to a delay in diagnosis [9]. Fracture diagnosis is typically based on a clinical review. The chief signs are swelling and decreased range of movement of affected limbs, and extreme pain in passive movements like changing diapers. Fractures can sometimes be identified incidentally during investigation for other causes, like neonatal respiratory distress [10].

The fractures during childbirth usually have a good prognosis with aggressive conservative management. The common treatment modalities to treat newborn femoral fractures are posterior limb splinting, spica cast application, gallows traction, Bryant's traction, and Pavlik harness, and all of them show good clinical and radiological improvement within three weeks. The use of the Pavlik brace in infants with femoral fractures is easier and justifiable rather than other conventional traction methods due to less skin irritation, dermatitis, and risk of sciatic nerve palsy [9]. Similar to Vellingiri et al. [11], we have used strapping involving the thigh and leg with minimal handling for the management of the fractured limb, as it has been reported in some studies that diaphyseal femoral fractures in neonates can be successfully treated with cast immobilization without the need for precise alignment of bony fragments [12].

The prognosis following timely intervention in femoral birth fractures is usually good without any residual limb length discrepancy or deformities in the future, as newborns show extraordinary signs of skeletal remodeling. In rare cases of extremely unstable epiphyseal detachment, surgery for osteosynthesis followed by growth monitoring is required throughout adolescence. Existing literature does not report complications like malunion or non-union in neonates with conservative treatment of femoral birth fracture [12]. In our case, signs of new bone formation around the fractured site were evident from the X-ray performed after three weeks of initiating treatment.

## Conclusions

Proper obstetric maneuvers and delivery protocols are necessary to decrease the incidence of birth fractures. Femoral shaft fracture is a rare outcome in cases of forced manipulation during difficult delivery. An orthopedic consultation and urgent X-ray are recommended on clinical suspicion of a fracture. Early detection and management with a cost-effective method, like strapping or casting of the affected limb, can heal the fracture without any residual deformity.
